# Toward attenuating the impact of arm positions on electromyography pattern-recognition based motion classification in transradial amputees

**DOI:** 10.1186/1743-0003-9-74

**Published:** 2012-10-05

**Authors:** Yanjuan Geng, Ping Zhou, Guanglin Li

**Affiliations:** 1 Key Lab of Health Informatics of Chinese Academy of Sciences (CAS), Shenzhen, China; 2 Institute of Biomedical and Health Engineering, Shenzhen Institutes of Advanced Technology, Chinese Academy of Sciences (CAS), 1068 Xueyuan Avenue, Nanshan District, Shenzhen, China; 3 Sensory Motor Performance Program, Rehabilitation Institute of Chicago, Chicago, IL, USA

## Abstract

**Background:**

Electromyography (EMG) pattern-recognition based control strategies for multifunctional myoelectric prosthesis systems have been studied commonly in a controlled laboratory setting. Before these myoelectric prosthesis systems are clinically viable, it will be necessary to assess the effect of some disparities between the ideal laboratory setting and practical use on the control performance. One important obstacle is the impact of arm position variation that causes the changes of EMG pattern when performing identical motions in different arm positions. This study aimed to investigate the impacts of arm position variation on EMG pattern-recognition based motion classification in upper-limb amputees and the solutions for reducing these impacts.

**Methods:**

With five unilateral transradial (TR) amputees, the EMG signals and tri-axial accelerometer mechanomyography (ACC-MMG) signals were simultaneously collected from both amputated and intact arms when performing six classes of arm and hand movements in each of five arm positions that were considered in the study. The effect of the arm position changes was estimated in terms of motion classification error and compared between amputated and intact arms. Then the performance of three proposed methods in attenuating the impact of arm positions was evaluated.

**Results:**

With EMG signals, the average intra-position and inter-position classification errors across all five arm positions and five subjects were around 7.3% and 29.9% from amputated arms, respectively, about 1.0% and 10% low in comparison with those from intact arms. While ACC-MMG signals could yield a similar intra-position classification error (9.9%) as EMG, they had much higher inter-position classification error with an average value of 81.1% over the arm positions and the subjects. When the EMG data from all five arm positions were involved in the training set, the average classification error reached a value of around 10.8% for amputated arms. Using a two-stage cascade classifier, the average classification error was around 9.0% over all five arm positions. Reducing ACC-MMG channels from 8 to 2 only increased the average position classification error across all five arm positions from 0.7% to 1.0% in amputated arms.

**Conclusions:**

The performance of EMG pattern-recognition based method in classifying movements strongly depends on arm positions. This dependency is a little stronger in intact arm than in amputated arm, which suggests that the investigations associated with practical use of a myoelectric prosthesis should use the limb amputees as subjects instead of using able-body subjects. The two-stage cascade classifier mode with ACC-MMG for limb position identification and EMG for limb motion classification may be a promising way to reduce the effect of limb position variation on classification performance.

## Background

Myoelectric signals recorded with electrodes on the skin surface overlying the residual arm muscles have been used in control of motorized upper-limb prostheses for several decades [1-23]. A significant improvement over the traditional EMG control method in myoelectric prostheses is the use of EMG pattern recognition based control strategy, which is grounded on the assumption that the patterns of EMG signals regarding the intended movements are consistent and repeatable. Most previous efforts focused on evaluating the capability of EMG pattern-recognition algorithms in identifying a number of motion classes in an ideal laboratory setting. Because of some disparities between laboratory investigation and practical use of a myoelectric prosthesis, it should be required to test the control performance in the conditions of the clinical setting before the myoelectric prosthesis systems can be clinically viable. Recently, the influences of some possible issues associated with clinical applications on the control performance of a multifunctional myoelectric prosthesis have come to the attention. To minimize the effect of unintended movements caused by motion misclassification during the real-time EMG pattern-recognition control, Simon et al. reported the use of decision-based velocity ramp that could attenuate movement speed after a change in classifier decision [17]. Their post-processing approach could provide a finer and smooth transition from current motion class to next identified one. In clinical use of a myoelectric prosthesis, misalignment inevitably occurs during prosthesis donning and doffing, resulting in a change of electrode locations contacted with skin. Young et al. investigated how the size of the electrode detection surface and the electrode orientation affected the robustness of EMG pattern-recognition based prosthesis control system with electrode shift [18]. While these reported progresses have been significantly made towards the clinical applications of EMG pattern-recognition based control, there are still some important disparities between the laboratory research results and the clinical performance that remain to be addressed before the multifunctional myoelectric prostheses are available for clinical use.

In most reported studies of EMG pattern recognition systems for multifunctional prosthesis control, subjects generally took a seated position with a tested arm resting on a plate surface such as chair arm or table and multi-channel EMG signals were acquired with a number of surface electrodes placed on either the muscles of forearm and hand for an able-bodied subject or the residual muscles for an upper-limb amputee. One portion of the acquired EMG data was used to train a classifier and then remaining portion was loaded into the trained classifier for calculating the offline classification accuracy in identifying a number of arm and hand movements [4-18,21-23]. With this experimental setting mode, high classification accuracies were often achieved since the training and testing EMG data could be consistently recorded in a constant position of the tested arm. However, this procedure would be different from the clinical application of a multifunctional myoelectric prosthesis, where the user’s arm position varies when he/she is going to activities of daily living.

In order to achieve the high classification accuracy of EMG pattern recognition approach for control of a multifunctional myoelectric prosthesis, it is required that the contraction of the targeted muscles could produce the repeatable EMG patterns for doing a movement. This is not the case in doing daily activities that would need the user’s arm position to be various. Keeping arm in different positions and performing a movement may require different forearm muscles to be contracted. Thus when doing identical movements in different arm positions, the arm muscles may generate disparate EMG patterns. Therefore, with a classifier trained by EMG recordings in one specific arm position, the EMG pattern changes may erode the classification accuracy of movements. This raises a question: whether does the variation of arm positions significantly affect the control performance of multifunctional myoeletric prosthesis? If the answer is yes, the following question is: how to reduce the impact of arm position variations?

Most recently, a study has been conducted by a research group to address these issues [19,20]. Their results showed that the variations in arm position associated with the clinical use of a myoelectric prosthesis could substantially impair the classification performance of EMG pattern recognition with an increase of average classification error from 3.8% to 18%. And they also proposed two possible solutions for reducing the effects of adverse arm positions on the motion classification accuracy of EMG pattern recognition. It is important to note that the reported results were achieved in the subjects with intact arms. It remains unclear whether the similar results could be achieved by arm amputees who are the final users of a myoelectric prosthesis, as no work has been done with this population. In this study, using transradial amputees as subjects we investigated the effect of diverse arm positions on the classification performance in identifying a number of classes of arm and hand movements involved in amputated arms. Two types of signals associated with muscle contractions, EMG acquired with surface electrodes and ACC-MMG measured with accelerometers, were used as prosthetic control signals for classifying motion classes. The sensitivity of EMG and ACC-MMG based pattern recognition for motion identification to adverse arm positions was investigated, respectively. And then three possible solutions were examined for the performance in attenuating the impact of arm position variation on classification performance. The outcomes of this study could aid the future development of practical multifunctional myoelectric prostheses for arm amputees.

## Methods

### Participants

Five subjects (1 female, 4 males) with unilateral transradial (TR) amputation aged from 22 to 43 years participated in the study. Their post-amputation times varied from 2 years to 10 years. The length of their residual forearms ranges from 5 cm to 14 cm (5cm, 12cm, 8cm, 14cm, 11.5cm). They wear either a myoelectric prosthesis or a cosmetic prosthesis everyday. The protocol of this study was approved by the Shenzhen Institutes of Advanced Technology Institutional Review Board, China. All subjects gave the written informed consent and provided permission for publication of photographs with a scientific and educational purpose.

### Experiments

**Figure 1:**
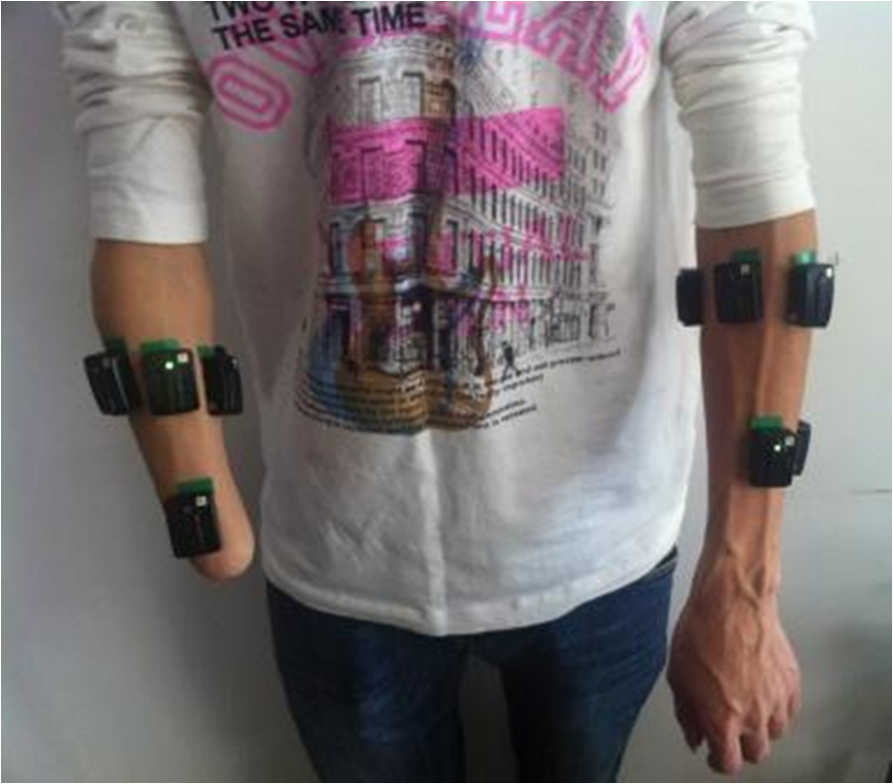
**Electrode placement.** Eight parallel-bar electrodes were placed on the skin surface of intact arm and amputated arm, respectively.

In this study a commercial wireless biological signal acquisition system (Delsys Inc, Boston, USA) was used to record surface EMG and ACC-MMG signals with eight sensors. Each sensor integrates one bipolar EMG electrode with one tri-axial ACC-MMG accelerometer, which could provide one-channel EMG recording and simultaneously one-location ACC-MMG recording. Note that the ACC-MMG signal, a three-dimensional measure of a sensor location, is composed of three sub-signals in x, y, and z axis, respectively. So with 8 sensors, 8-channel EMG signal recordings and 8-position ACC-MMG signal recordings with 24-channel could be simultaneously obtained. For each subject, six of the eight bipolar EMG sensors were placed around the apex of the muscle bulge, 1–2 cm distal to the elbow crease and another two sensors were placed on the distal end over flexor muscle and extensor muscle, respectively, as illustrated in Figure 1. Each sensor of the Delsys system is integrated with a built-in tri-axial accelerometer, so EMG and ACC-MMG signals can be recorded simultaneously with the hybrid sensors. For the sake of comparison, in the experiments TR subjects were asked to perform arm and hand movements simultaneously using their amputated arm and intact arm. Also, eight sensors were placed on intact arms of each subject at almost same locations as on amputated arms. Totally, 16-channel EMG and ACC-MMG signals were acquired with all the 16 sensors from both arms during experiments.

**Figure 2:**
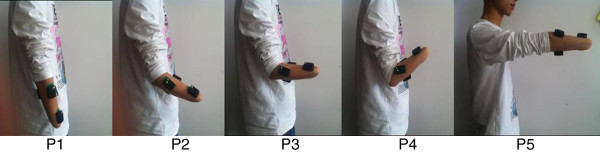
Five limb positions considered in the study.

For each subject, experiment was comprised of five sets. In each set, the subject was instructed to perform seven classes of frequently used forearm movements at a moderate and repeatable force level with his/her arms in one of five typical arm positions considered in the study. The seven classes of forearm movements were wrist flexion (WF), wrist extension (WE), wrist supination (WS), wrist pronation (WP), hand open (HO) and hand close (HC), plus a “no movement” (NM) class. Five typical arm positions commonly used in daily life activities (Figure 2) were considered in this study for evaluating the possible effects of arm position variation on motion classification performance.

Subjects were prompted to elicit contractions corresponding to the target motion class displayed in a video. Each movement contraction was sustained for 4 s to generate myoelectric signals and the rest time between subsequent contractions was 5 s. Each of seven movements was repeated 10 times per set. Thus each set produced 40-sec EMG and ACC-MMG recordings per movement. To avoid muscle and mental fatigue, subjects were allowed to have a rest for 10–15 minutes between sets. During experiments EMG and ACC-MMG data were simultaneously acquired with a sampling rate of 4000 Hz for EMG signals and 296.3 Hz for ACC-MMG signals. With 8 EMG-ACC-MMG hybrid sensors, the 8-channel EMG signal recordings and 24-channel (3×8) ACC-MMG signal recordings (each accelerometer could get three dimensional measures of position) could be simultaneously obtained from residual arm and intact arm, respectively.

### Data pre-processing and feature extraction

EMG and ACC-MMG signals were acquired with the maximum sampling rates of the commercial bioelectric signal acquisition system (*Delsys Inc*.). Considering that the major power (about 95%) of surface EMG signals is often below 400–500 Hz, the EMG signal recordings were down-sampled to 1 kHz to simplify data processing. To remove the slow variations in the EMG signals caused by the motion artifacts such as electrode shift and cable movement, the EMG signal recordings were digitally filtered with a five-order Butterworth high-pass filter at a 5 Hz cut-off frequency.

In the study, shifting analysis window with a time length of 150 ms and an increment of 100 ms (50-ms overlapping) was used for feature extraction [14,21,22]. The EMG and ACC-MMG data recordings corresponding to each movement were divided into a series of analysis windows and then the features were extracted from each analysis window. For EMG signals, the commonly used four time-domain (TD) statistics that have showed the suitable representation for EMG signals [8,14,19-22], mean absolute value, number of zero crossings, waveform length and number of slope sign changes, were adopted in the study. For ACC-MMG signals, three time-domain features (mean absolute value, variation and maximum value) were used to represent ACC-MMG patterns.

### Pattern recognition analysis

A simple linear discriminant analysis (LDA) [7,8,14-23] was used in the study to build a classifier for the classification of motion classes. More complex classifiers such as artificial neural network, hidden Markov model, and fuzzy logic classifiers may be considered, but it has been reported in previous works [8,23] that a LDA classifier would not compromise the accuracy of motion classification. Moreover, compared to other complex classifiers, LDA classifier is much simpler and faster to implement. Thus using a simpler LDA classifier would be computationally efficient for real-time myoelectric prosthesis control.

For each subject, the features from the first half of EMG or ACC-MMG recordings (20-sec) were concatenated as the train data set to train a LDA classifier; the features from the second half of EMG or ACC-MMG recordings (20-sec) were also combined together as the test data set to estimate classification performance of the trained classifier. Classification error was used to evaluate the motion identification performance in this study, which is defined as:

(1) $$\frac{\text{Number of incorrectly classified samples}}{\text{Total number of testing samples}}\times 100\%$$

The average value of classification errors in identifying all seven classes of movements was calculated as the overall classification error for a subject.

For each subject, in order to assess the sensitivity of EMG pattern recognition for motion classification to the five arm positions, a LDA classifier was built for each arm position, totally producing five single arm position classifiers. Then the five test feature sets corresponding to the five arm positions were fed into each of the five trained classifiers to calculate the classification errors, respectively, resulting in an overall classification error matrix (5×5) in which the diagonal elements represented the intra-position classification errors and the non-diagonal elements were inter-position classification errors. Similarly, an overall classification error matrix also was obtained for each subject when ACC-MMG signals were used for motion classification.

**Figure 3:**
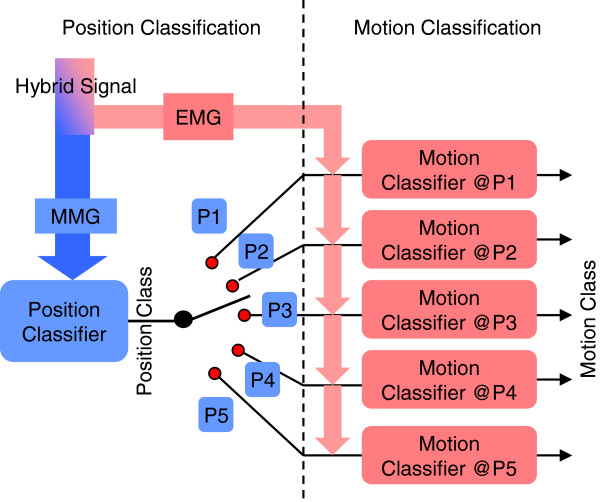
Two-stage Cascade Classifier.

With an attempt to attenuate the impact of arm position variation on classification performance, three possible solutions were proposed in the study as follows:

1) *Training a classifier with both EMG and ACC-MMG data from a single position* - By training a classifier with the combination of EMG and ACC-MMG signal recordings at each of five arm positions, we looked for if involving more training information associated with arm movements could significantly increase the robustness of the classifier against the impact of arm position variation.

2) *Training a classifier with EMG data from multiple arm positions -* In order to investigate if using EMG data from two or more arm positions to train a classifier could substantially reduce the influence of arm position variation on the classification performance, a LDA classifier was trained with the concatenated EMG data from multiple arm positions. With five arm positions, the possible combination number was 10 for two- and three-position, 5 for four-position, and 1 for five-position combinations. Totally, 26 multi-position classifiers would be built. And then each of trained multi-position classifiers was tested using the concatenated EMG data from all the five positions.

3) *Using a cascade classifier* - The cascade classifier was composed of two sequential classifiers, as shown in Figure 3. The first stage was a position classifier that was trained with ACC-MMG recordings and used to identify the position of subject’s arm. The second stage was composed of five movement classifiers that were used to classify motion classes. Each of the five movement classifiers corresponded to a specific arm position and was trained with the EMG data from all seven classes of movements performed in the arm position. The position classifier was first used to get the arm position for the selection of a movement classifier corresponding to the arm position and then the selected movement classifier was used to get the class of movements.

### Channel reduction analysis

Generally speaking, using more signal recording channels could get more motion information for better performance of movement classification, but may increase the complexity of computation and analysis that may lead to slow discrimination response. A pilot analysis was performed in this study to investigate the feasibility of using a reduced number of electrodes without compromising classification accuracy. With a straightforward exhaustive search algorithm [14] we also investigated the relationship between the number of EMG and ACC-MMG channels and the classification errors, respectively. Channel number reduced from 8 to 1 with a decrement step of 1. All possible combinations for a reduced number of channels were evaluated by classification error for the seven movement classes. The channel combinations that produced the lowest classification error for each number of channels were considered as the “optimal” channel configurations.

### Statistical analysis

In this study, a paired *t*-test was used to assess the statistical difference between the means of compared classification errors and the level of statistical significance was set to *p<0.05*.

## Results

### Effect of arm position variation in amputated arm

**Figure 4:**
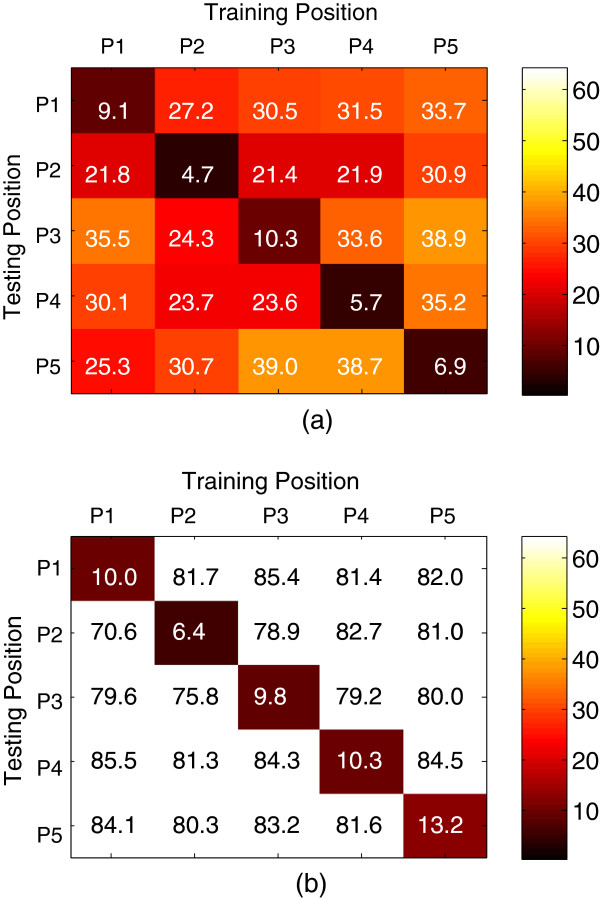
**Motion classification error matrices.** Each column represents the limb position from which training data comes, and each row denotes the limb position from which testing data comes. **(a)** using EMG as classifier’s input. (**b**) using ACC-MMG as classifier’s input.

The average overall classification error matrix over all the five subjects was calculated for their amputated arms and showed in Figure 4(a) when only using EMG signals and in Figure 4(b) when only using ACC-MMG signals. It can be seen from Figure 4 that using either EMG or ACC-MMG as movement classification signal could produce similar intra-position classification performance, but distinct inter-position classification performance. The average intra-position classification error across all the five arm positions and the five subjects was around 7.3±2.3% for EMG and 9.9±2.4% for ACC-MMG, which were no significant difference (*p*-value=0.11). However, the ACC-MMG signals had much higher inter-position classification error with an average value of 81.1±1.5% over the arm positions and the subjects, in comparison of the EMG signals with an average value of 29.9±3.2%; the difference was significant (*p*-value<0.01). This suggests that ACC-MMG signals are much more sensitive to arm position variation in comparison to EMG signals.

### Effect of arm position variation in intact arm

**Figure 5:**
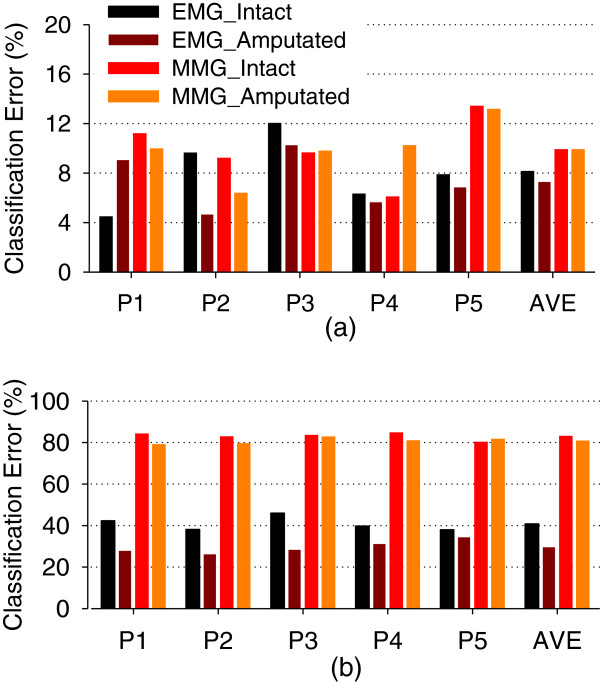
**Intra-position Motion Classification Comparison.** Intra-position and Inter-position motion classification errors. The training data and testing data come from same limb positions. EMG and ACC-MMG were used as classifier’s inputs for intact limb and amputated limb, respectively. **(a)** Intra-position motion classification error. **(b)** Inter-position motion classification error.

The classification errors over the five subjects also were calculated for their intact arms. Figure 5 illustrates the average intra-position classification errors versus the five arm positions in the four cases, intact arm with EMG and ACC-MMG as well as amputated arm with EMG and ACC-MMG. With EMG signals, it can be surprisingly seen from Figure 5(a) that all the intra-position classification errors from amputated arm were lower than those from intact arm except that in the arm position *P1*. The average intra-position classification error over the five arm positions from amputated arm was about 1.0% lower than that from intact arm, as shown in the most right column of Figure 5(a). Similarly, the amputated arm could achieve better inter-position classification performance with EMG signals than intact arm, but the difference was significant (*p*-value<0.01). The average inter-position error across all arm positions and subjects were 29.9±3.2% for amputated arm and 40.9±3.4% for intact arm (the most right column of Figure 5(b)).

### Single-position classifier trained with both EMG and ACC-MMG signals

With an attempt of attenuating the effect of arm position variation on classification performance, single-position classifiers were trained and tested with the information of EMG and ACC-MMG combinations. Table 1 summarized the average classification errors over all the subjects in five positions of an amputated arm. With EMG plus ACC-MMG signals, the average intra-position classification error across all subjects decreased to 5.6 ± 1.0% from 7.3 ± 2.3% when only using EMG and from 9.9 ± 2.4% when only using ACC-MMG. The average inter-position error was 73.9 ± 4.8% when using EMG plus ACC-MMG, 29.9 ± 2.9% when using EMG, and 81.0 ± 1.3% when using ACC-MMG. Totally, the average classification error across all positions and subjects was 60.2% for the classifier trained with EMG and ACC-MMG combination.

**Table 1:** Motion Classification Performance Comparison of Three Single-Position Classifiers with Different Input

	**Intra-Position**			**Inter-Position**		
**Input Type**	**Hybrid**	**EMG**	**ACC-MMG**	**Hybrid**	**EMG**	**ACC-MMG**
P1	5.0 %	9.1%	10.0%	79.5%	28.2%	79.3%
P2	4.1 %	4.7%	6.4%	67.5%	26.5%	79.8%
P3	6.7 %	10.4%	9.8%	71.0%	28.6%	83.0%
P4	6.0 %	5.7%	10.3%	77.4%	31.4%	81.1%
` P5	6.1 %	7.0%	13.2%	73.9%	34.7%	81.8%
AVE±STD	5.6±1.0%	7.3±2.3%	9.9±2.4%	73.9±4.8%	29.9±2.9%	81.0±1.3%

The classification errors are averaged over all subjects for their amputated arms

### Multi-position classifier trained with EMG

**Figure 6:**
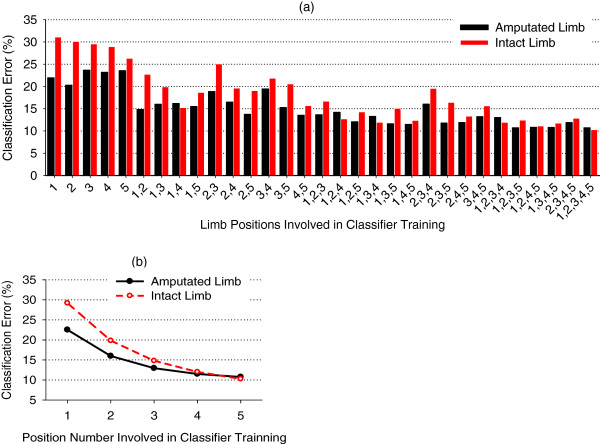
**Multi-position Classifier.** Multi-position classifiers. With training data from 1 to 5 arm positions. **(a)** Motion classification errors with respect to all possible position combinations. **(b)** Averaged classification error as position number range from 1 to 5.

Compared to EMG signals, ACC-MMG signals are much more sensitive to arm position variation (Figures 4 and 5). Thus multi-position classifiers were trained only using EMG data from multiple arm positions (P1-P5) and tested using EMG data from all five arm positions. The average classification errors of 31 movement classifiers (26 multiple positions plus 5 single position) from the amputated arms of all five subjects were calculated and are presented in Figure 6(a). For the sake of comparison, the average error bars from subjects’ intact arms were also calculated and are plotted in Figure 6(a). Generally speaking, the average classification error gradually decreased along with more arm positions including in classifier training. When the EMG data from all five arm positions were involved in the training set, the average classification error reached a minimum value of around 10.8% for the amputated arm and 10.3% for the intact arms, as shown in Figure 6(b). It is noteworthy that in most of the 31 motion classifiers, the amputated arms had a low classification error, in comparison with the intact arms.

### Two-stage cascade classifier

#### Arm position classification

**Figure 7:**
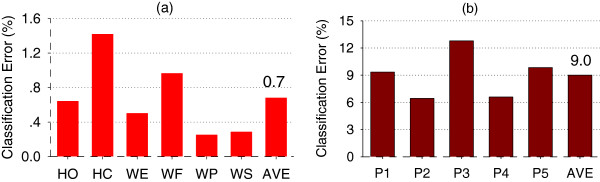
**Two-stage Cascade Classifier. (a)** Stage 1: Position classification using ACC-MMG **(b)** Stage 2: Motion classification using EMG.

The first stage of the cascade classifier was a position detection classifier trained by ACC-MMG data. The position classification errors for the detection of five arm positions are showed in Figure 7(a). The average position classification error across all subjects and six forearm motion classes was 0.7% ± 0.4% for amputated arms.

#### Movement classification with a specific arm position

The second stage had five movement classifiers corresponding to the five arm positions. For a specific arm position selected with the result of the position classifier, EMG data test sets from all seven classes of movements were used to calculate the classification errors. Figure 7(b) illustrates the average classification errors across all five subjects and seven motion classes in five arm positions. It can be seen from Figure 7(b) that the cascade classifier had an average classification error of 9.0% over all five arm positions.

### Channel number reduction

**Figure 8:**
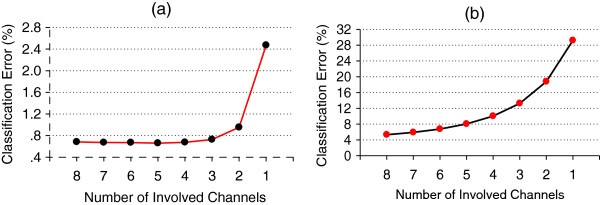
**Channel Reduction Analyses. (a)** Position classification error with channel reduction by step 1 when using ACC-MMG. **(b)** Motion classification error with channel reduction by step 2 when using EMG.

For a position classifier trained with ACC-MMG data, reducing ACC-MMG channels from 8 to 2 only increased the average position classification error across all five arm positions from 0.7% to 1.0% in amputated arm, as shown in Figure 8(a). For the movement classifier trained with EMG data, using five optimally selected channels produced an average classification error of 8.0% over all subjects and seven motion classes, compared to 5.3% with all the 8 channels as shown in Figure 8(b).

## Discussion

The effect of the change of arm positions in doing different daily tasks on the performance in classifying arm movements was investigated in the study. Although the recent studies have been done to evaluate the effect of arm position variation on the classification performance [19,20], their findings were obtained from able-body subjects, not limb amputees who are the final users of a myoelectric prosthesis. With the loss of arm, when doing same movements at a specific arm position, the residual muscles of an amputated arm may give different EMG patterns from the muscles of an intact arm. This requests a study to be implemented in amputees for assessment of the influence of arm position changes in the clinical use of a myoelectric prosthesis. In this study we used transradial amputees as subjects to investigate the impact of five typical arm positions and analysed the performance of three possible solutions in increasing the robustness of myoelectric control system against arm position variation. Besides the commonly used EMG, ACC-MMG was also used in the study as an additional signal for performing motion classification. ACC-MMG is a measure of the oscillations generated by muscle contractions and propagated through the fat and skin and is non-invasively recorded using accelerometers on the skin surface. As an alternative related to muscle contraction, ACC-MMG has been used in a couple of previous studies for control of external powered prostheses [24,25]. Most recently, Zhang et al. realized the gesture language identification with hybrid EMG and ACC-MMG [26].

For a single-position classifiers trained at a specific arm position, EMG and ACC-MMG recordings from amputated arms yielded similar average intra-position classification errors (7.3% versus 9.9%) in classifying seven classes of arm and hand movements, but significantly different average inter-position classification errors (around 30% versus 81%). The significant difference between intra-position and inter-position classification errors indicate that the classification performance of either EMG classifier or ACC-MMG classifier could be substantially affected by the arm position changes, which is consistent with the results of the reported able-body subject study [19,20]. The significant impact of arm positions may be due to the EMG or ACC-MMG pattern changes that were caused mainly by muscle contraction variations required for holding arm in a spatial position. This indicates that a classifier trained in a specific arm position would perform well in classifying the movements done in the position, but may be not sufficient too in other arm positions.

With a quite high average inter-position classification error, ACC-MMG recordings should be much more sensitive to arm position variations; this indicates that ACC-MMG is good at classification of different arm positions, but poor at motion classification. It is noteworthy that EMG recordings from amputated arms produced the low average intra-position (about 1% low) and inter-position (about 10% low) classification errors, in comparison to those from intact arms. This may be the most important finding of the current study. Note that most previous studies [8,20] used able-bodied people as the subjects to assess the feasibility and performance of pattern-recognition algorithms using EMG signals from forearm muscles. This should be reasonable and necessary for a simple goal of comparing classification accuracy of different pattern recognition algorithms, but the findings achieved from able-bodied subjects or an intact limb might not be simply deduced to amputees or an amputated limb [14]. After arm amputation, a residual arm is shorter and lighter than an intact arm in same subject. Thus less impact of arm position variation in amputated arms may be due to less gravitational force needed to stabilize a remaining arm in a spatial position in comparison with an intact arm. Thus this interesting finding suggests again that the investigations related to some practical issues of multifunctional prosthesis system applications should be conducted in limb amputees instead of able-bodied subjects.

With an attempt to look for a suitable method for attenuating the influence of arm position variations on EMG pattern-recognition prosthesis control, three possible solutions were examined in this study. The first solution was to build a single-position classifier trained with the combining information of EMG and ACC-MMG. This hybrid classifier yielded lower average intra-position classification error (5.6%) than the EMG classifier (7.3%) or the ACC-MMG classifier (9.9%), but produced a quite high average inter-position classification error (around 74%). The lower intra-position classification errors indicate that including more information representing muscles activities into training data set could generally get better classification performance, which is consistent with the reported study [21]. The high inter-position classification errors would be attributed to the high sensitivity of ACC-MMG signals to arm position. These results obviously show that this solution would be an insufficient method to reduce the impact of arm position variations in multifunctional myoelectric prosthesis systems.

Another solution considered in this study was to train a multi-position classifier. Since ACC-MMG is very sensitive to arm position variation, the multi-position classifier was only trained with EMG. By adding EMG data from more arm positions into training set, the effect of arm positions on EMG patterns could be involved in the trained classifier, which would increase the classifier’s generalization or robustness. This was proved by the experimental results of the study. The more the arm positions in EMG training set were involved, the more the classification performance was improved (Figure 6). When the EMG data from all five arm positions were involved in the training set, the average classification error reached a minimum value (10.8%) for the amputated side.

The third solution examined in the study was a two-stage cascade classifier. Taking the high sensitivity of ACC-MMG to arm positions and EMG to movements, the position classifier was trained with ACC-MMG data and the movement classifier was trained with EMG data. Table 2 summarized the average motion classification error over all subjects by means of these three solutions above mentioned, in comparison with that across all five arm positions when only EMG or ACC-MMG signals were used. The results achieved in the study show the two-stage cascade classifier solution was the best one among the three possible methods. This suggests that the cascade classification strategy may be promising for the accurate and reliable control of EMG pattern-recognition based prosthetic systems in practical use. Note that using two-channel ACC-MMG signals instead of eight-channel ACC-MMG only increased the position classification error slightly (from 0.7% to 1%). Small number of channels would simplify the myoelectric prosthesis system and reduce the cost and power consumption.

**Table 2:** Motion Classification Performance Comparison

**EMG**	**ACC-MMG**	**Solution 1**	**Solution 2**	**Solution 3**
25.4±6.5%	66.8±1.4%	60.2±0.7%	10.8±4.5%	9.0±4.7%

The motion classification performance of three solutions with average classification errors over all subjects for their amputated arms, in comparison with traditionally used pattern recognition method with classification error averaged over all subjects and all five arm positions.

Note that the classification performance was evaluated by the classification errors that were calculated by post-processing EMG recordings (offline) and was not a direct measure of real-time performance in clinic. Generally speaking, the high offline classification errors may decay the accuracy and reliability of a multifunctional prosthesis control in real time application. Three real-time performance measures have been proposed [22] to gain insight into the feasibility of clinically implementing EMG pattern recognition-based controllers for arm amputees. Using these real-time performance metrics, future investigations will be conducted to further validate the feasibility and performance of the proposed methods in this study. In addition, with a purpose of evaluating the effect of arm position variation on the classification performance in multifunctional myoelectric prostheses, five typical/representative arm positions, which are parallel to the sagittal plane, were chosen in the study. Besides these five arm positions, more arm positions such as those in transverse plane and coronal plane would be probably involved in some daily activities. It is a limitation that no arm positions in other planes such as transverse and coronal plane were involved in this study, which will be considered in our further studies to see if the arm position changes in other planes has different effect on the classification performance.

## Conclusions

The current study used the transradial amputees as subjects who are the final user of myoelectric prostheses to assess the effects of arm position variation on EMG and/or ACC-MMG pattern-recognition based motion classification in limb amputees and evaluated the performance of three proposed solutions in reducing the impact of arm positions. For amputated arms, the average inter-position error of EMG classification across all the five arm positions and the five subjects was around 22% higher than the average intra-position error. This indicates that the performance of EMG pattern-recognition based method in classifying movements strongly depends on arm positions. This dependency is stronger in intact arm than in amputated arm, which suggests that the investigations associated with practical use of a myoelectric prosthesis should be conducted with the limb amputees as subjects instead of able-body subjects. Using eight-channel ACC-MMG signals as input of arm position classifier could achieve an average classification error as low as 0.7% across five arm positions and five subjects; even though using two-channel ACC-MMG signals, the position classification error slightly increased to 1%. Thus ACC-MMG signals would be very suitable for the arm position identification. With ACC-MMG and EMG data as the input signals of arm position and movement classifier, respectively, the two-stage cascade classifier could obtain the best performance in attenuating the impact of arm position variation among three proposed solutions. This suggests that the cascade classification strategy may be promising for the accurate and reliable control of EMG pattern-recognition based prosthetic systems in practical use.

## Competing interests

The authors declare that they have no competing interests.

## Authors' contributions

YG performed experiment design, subject recruitment, data collection and analysis, interpretation of the results, and drafting of the manuscript, PZ was involved in interpretation of the results and the manuscript preparation, GL oversaw the study and was involved in each stage of the study, including critical revision of the manuscript. All authors read and approved the final manuscript.
